# Pityriasis Lichenoides: Comparison of Two Pediatric Patients and Its Relationships With Vaccines and Infections

**DOI:** 10.1002/ccr3.73033

**Published:** 2026-06-25

**Authors:** Gregorio Serra, Deborah Bacile, Elisabetta Di Leto, Ornella Iacono Fullone, Chiara Martorana, Ilaria Pirrone, Mario Tumminello, Giovanni Corsello

**Affiliations:** ^1^ Department of Health Promotion, Mother and Child Care, Internal Medicine and Medical Specialties “G. D'Alessandro” University of Palermo Palermo Italy

**Keywords:** antigenic stimuli, COVID‐19, group A streptococcus, PLEVA, skin lesions

## Abstract

Pityriasis lichenoides (PL) is a benign, self‐limiting inflammatory condition, characterized by the appearance of papular, scaly, or necrotic lesions. Despite the rare incidence, clinicians should suspect its diagnosis in the presence of specific skin lesions and epidemiological contexts, as suggested by its association with infections and/or vaccinations.

## 1 Introduction

Pityriasis lichenoides (PL) is a benign, self‐limiting inflammatory condition of unknown etiology. It is an uncommon disease, which nevertheless frequently affects children. It is characterized by the appearance of papular, scaly, or necrotic lesions. PL is typically classified into three forms, considered the spectrum of the same disease: pityriasis lichenoides et varioliforme acuta (PLEVA), chronic PL (PLC), and febrile ulceronecrotic Mucha‐Habermann disease (FUMHD) [1].

It is currently considered a lymphoproliferative disorder secondary to antigenic, bacterial, viral, or parasitic stimulation, as suggested by its presentation in outbreaks. In addition, some vaccines such as MMR (measles‐mumps‐rubella), influenza, and recently COVID‐19 have been associated with the onset of PL [1].

In this article, two patients with PLEVA are reported and compared. Their main clinical characteristics are described, documenting for each one a different pathogenetic correlation. The aim of our report is to highlight the need for an accurate anamnestic approach and a detailed evaluation of the typical skin lesions over time, to ensure the correct and timely recognition of this rare condition and to guide its appropriate treatment and follow‐up.

## 2 Case History

**FIGURE 1:**
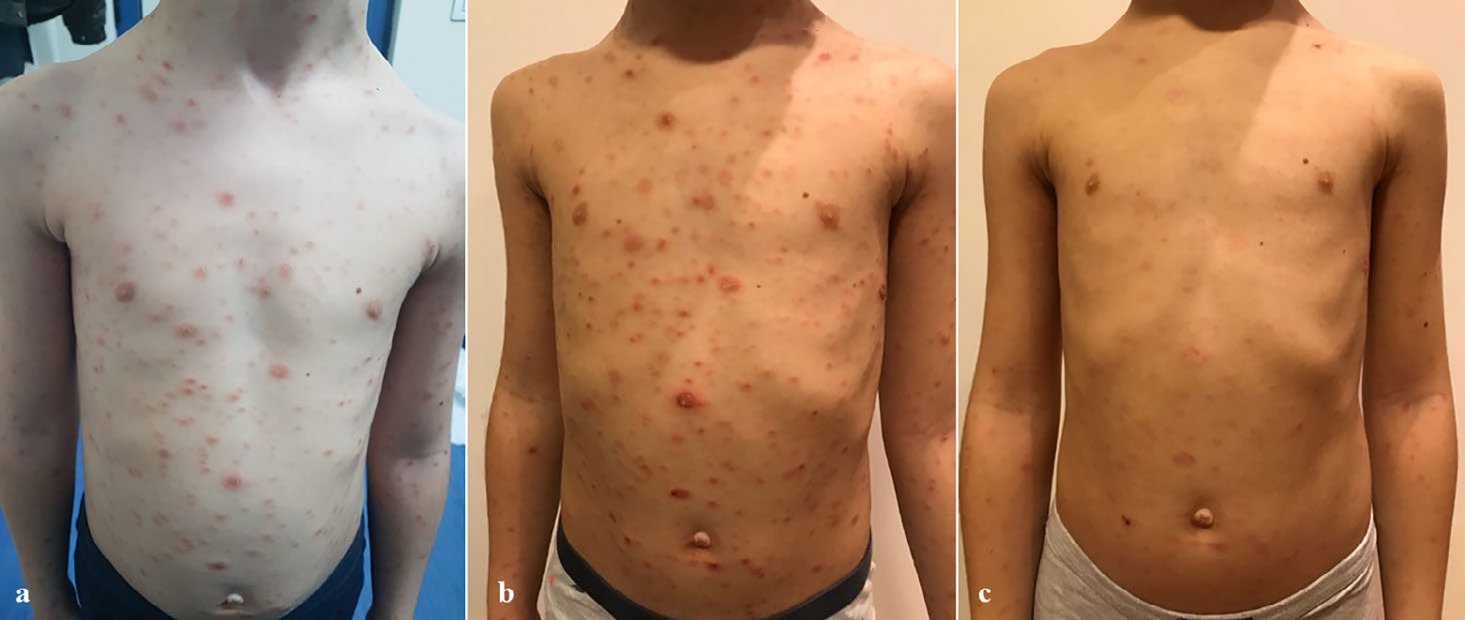
Patient 1. Clinical evolution of PLEVA lesions. (a) Multiple erythematous‐brown papules distributed on the trunk. (b) Progressive increase in the number of papules with development of purpuric discoloration and central adherent scale‐crust formation. (c) Marked clinical improvement with residual post‐inflammatory changes after 6 weeks of treatment.

Patient 1 is a 6‐year‐and‐10‐month‐old male child, who was brought to the observation of the family pediatrician in Palermo, Sicily (Italy), due to the onset of skin manifestations starting at the end of December 2021. The eruption initially consisted of two erythematous‐brown papules located on the anterior chest and proximal left upper limb. According to the parents' history, during the following 5 days the lesions rapidly increased in number and progressively spread to the trunk and upper extremities. Individual lesions evolved into multiple discrete and confluent erythematous‐to‐purpuric papules measuring approximately 3–4 mm in diameter, several of which developed central adherent scales and hemorrhagic crusts. Dermatological examination performed approximately 20 days after onset revealed disseminated papulo‐necrotic lesions at different stages of evolution, predominantly involving the trunk and upper limbs (Figure 1a,b). No mucosal involvement, fever, pruritus, or systemic symptoms were present. The remaining physical examination was normal. The past medical history was not significant, except for SARS‐CoV‐2 infection contracted 14 months earlier. Moreover, the parents reported that the child was vaccinated at the beginning of December 2021 against influenza virus and that, 20 days later, he received the first dose of COVID‐19 vaccine (BNT162b2, Pfizer‐BioNTech). Hematochemical tests, including inflammation indices, detected normal results; the infectious disease screening (hepatitis B and C, EBV, CMV, toxoplasmosis, parvovirus B19, HIV) was also negative, with a serological profile showing the recent vaccination (absent anti‐nucleocapsid antibodies, and positive anti‐SARS‐CoV‐2‐RBD Spike, 10 days after vaccine administration).

In the clinical suspicion of PLEVA, a therapy with topical hydrocortisone and oral azithromycin was started (the latter at a dose of 250 mg/day for five consecutive days/week, and repeated for two further cycles, every other week). The child was re‐examined after 6 weeks. Most of the lesions had regressed (Figure 1c).

**FIGURE 2:**
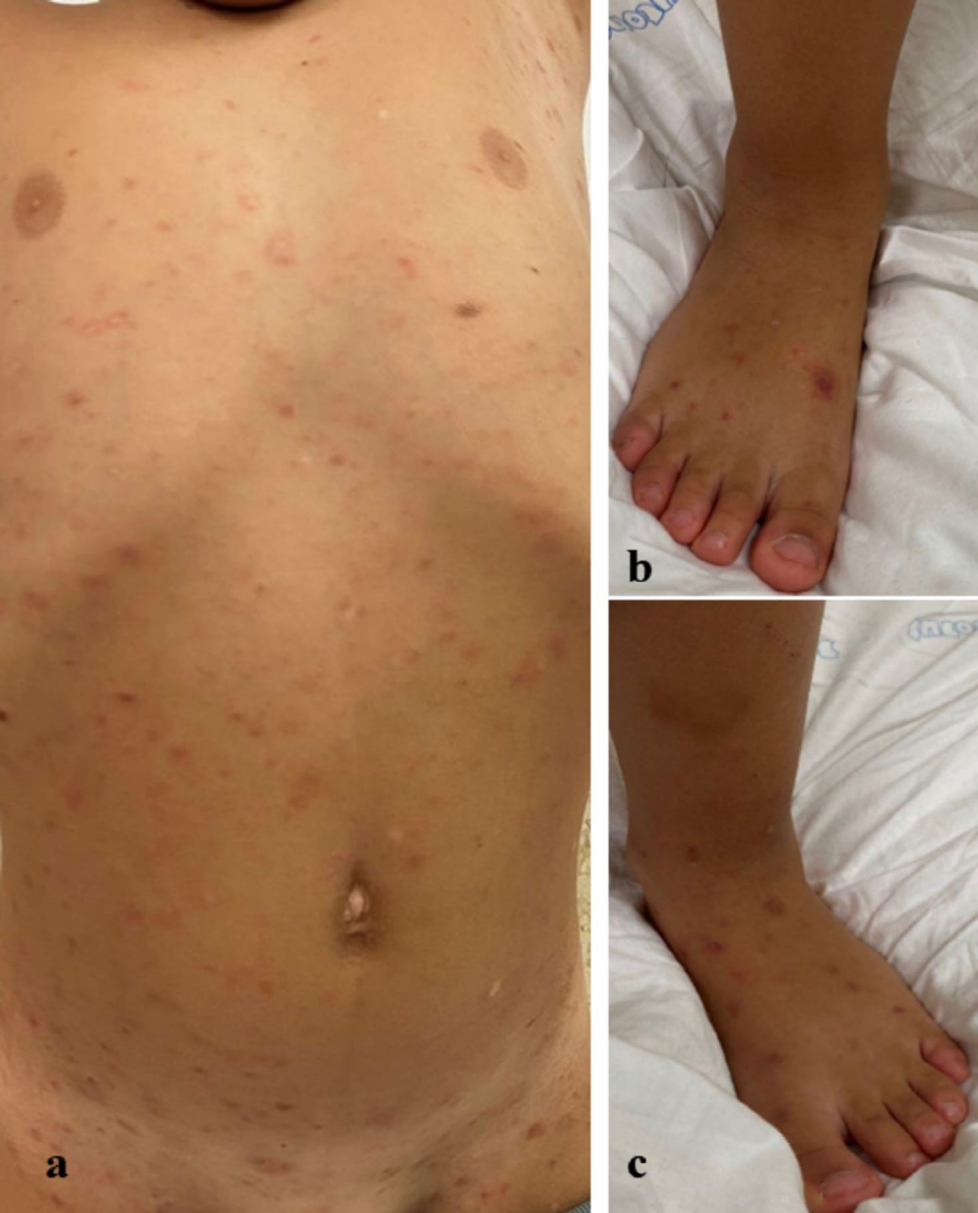
Patient 2. Polymorphic papular eruption consistent with PLEVA. (a) Truncal lesions showing erythematous and purpuric papules. (b, c) Lesions on the extremities demonstrating central necrosis and crust formation, with coexistence of lesions at different stages of evolution.

Patient 2 is a 5‐year‐old male child, who was taken to the pediatric emergency room of the Children's Hospital of Palermo, Sicily (Italy), due to the appearance, for about 5 days, of a slightly itchy micro‐papular rash. On the advice of the family pediatrician, antihistamine therapy was administered, up to that moment, without any benefits. The past clinical history was unremarkable. Physical examination revealed a body temperature of 37.4°C and numerous erythematous papules distributed over the trunk, extremities, and flexural areas. Several lesions showed purpuric discoloration, while others exhibited central necrosis and crust formation, resulting in a polymorphic eruption characterized by lesions at different evolutionary stages (Figure 2a–c). Mild pharyngeal erythema with tonsillar exudates was present. No mucosal lesions, lymphadenopathy, or other systemic abnormalities were detected. Laboratory tests, including complete blood count, liver and kidney function, and inflammation indices disclosed no abnormalities, as well as the infectious disease screening (major and minor hepatotropic viruses, Toxoplasma, Parvovirus B19, HIV), with the exception of the pharyngeal swab for group A beta‐hemolytic streptococcus (GAS), which conversely resulted positive.

In the clinical suspicion of PLEVA, likely secondary to the bacterial infection, a therapy with oral amoxicillin (50 mg/kg twice/day for 7 days) and topical hydrocortisone was started. The child showed a progressive regression of the lesions, which disappeared definitively after about 1 month. The long‐term follow‐up documented neither recurrence of skin manifestations, nor signs or symptoms affecting other organs and systems.

Histopathological examination was not performed in either patient. In both cases, the diagnosis of PLEVA was considered clinically supported by the characteristic morphology and distribution of the lesions, their evolution through different developmental stages, the exclusion of alternative infectious and systemic conditions, and the favorable clinical outcome. Although skin biopsy was discussed as a potential diagnostic procedure, the parents declined histopathological examination because of the invasive nature of the procedure. We acknowledge that the absence of histopathological confirmation represents a limitation of the present report, although the overall clinical findings were highly suggestive of PLEVA.

## 3 Differential Diagnosis

The diagnosis of PLEVA is primarily clinical and requires differentiation from several papulo‐necrotic, papulo‐squamous, infectious, inflammatory, and lymphoproliferative disorders [2, 3]. The main differential diagnoses considered in our patients were:

- Varicella: excluded because of the absence of vesicular lesions, systemic symptoms, and the typical centripetal distribution. Although lesions at different stages of evolution were present, the morphology was predominantly papular, purpuric, and necrotic rather than vesicular.
- Guttate psoriasis: particularly considered in Patient 2 because of the concomitant group A streptococcal infection. However, the lesions lacked the characteristic diffuse erythematous scaling pattern of guttate psoriasis and instead evolved toward purpuric and necrotic lesions with central crust formation.
- Pityriasis rosea: excluded because of the absence of a herald patch and the lack of the typical distribution along cleavage lines (Christmas‐tree pattern) [4, 5].
- Gianotti–Crosti syndrome: considered unlikely because of the predominant truncal involvement and the presence of purpuric and necrotic lesions, which are not characteristic features of this condition.
- Lymphomatoid papulosis: considered because of the papulo‐necrotic appearance of some lesions. However, this disorder usually presents with recurrent crops of larger papules and nodules, frequently showing ulceration and a chronic relapsing course, features that were absent in our patients.
- Langerhans cell histiocytosis: considered unlikely because of the acute onset, benign clinical course, complete resolution of lesions, and absence of systemic involvement.
- Secondary syphilis, disseminated herpes simplex infection, and arthropod bite reactions: excluded on the basis of clinical presentation and laboratory investigations.

The diagnosis of PLEVA was, conversely, supported by the following findings:

- abrupt onset of erythematous papules;
- evolution of lesions toward purpuric and necrotic papules with central scale‐crust formation;
- coexistence of lesions at different stages of development;
- predominant involvement of the trunk and proximal extremities;
- absence of significant systemic manifestations;
- exclusion of major infectious, inflammatory, and proliferative differential diagnoses;
- favorable clinical response to treatment and spontaneous tendency toward resolution.

Histopathological examination was not performed in either patient because skin biopsy was declined by the parents owing to the invasive nature of the procedure. Nevertheless, the overall clinical presentation, lesion evolution, exclusion of alternative diagnoses, and clinical follow‐up strongly supported the diagnosis of PLEVA [6].

## 4 Conclusion and Results

Pityriasis lichenoides is a polymorphic dermatitis due to its clinical appearance, duration, and unpredictable eruption of lesions. It is typically classified into three forms—acute (PLEVA), chronic (PLC), and febrile ulceronecrotic (FUMHD)—which represent the opposite poles of a broad spectrum of expression of the same disease [1]. PLEVA is characterized by the initial presentation of papular lesions or erythematous papules measuring 3–5 mm in diameter with purpuric, scaly, or necrotic evolution, diffusely distributed with predominance on the trunk, proximal limbs, and flexural areas. The lesions are generally asymptomatic and spare the face and mucous membranes. In some cases, lesions with necrotic‐hemorrhagic evolution may heal over several weeks leaving varioliform scars and may be associated with pruritus or burning sensations [1]. PLC presents as a persistent and usually asymptomatic eruption composed of erythematous and scaly papules that may be hyperpigmented or hypopigmented, often with a relapsing course lasting several months. Mucha‐Habermann disease represents a rare and severe variant of PLEVA, characterized by rapid onset of necrotic skin lesions accompanied by fever and multisystem involvement, including respiratory, gastrointestinal, neurological, cardiac, hematological, and joint manifestations. Progression to febrile ulceronecrotic Mucha‐Habermann disease (FUMHD) may occur within days or weeks, and may occasionally represent the initial presentation of PL [7].

Histologically, PL shows variable features depending on disease stage, including epidermal spongiosis, necrotic keratinocytes, dermal lymphocytic infiltrate, and focal parakeratosis [8]. FUMHD may share histopathological features with epidermotropic cytotoxic T‐cell lymphoma, raising concerns for potential overlap or malignant transformation in severe cases [9, 10].

The etiology of pityriasis lichenoides remains unknown; however, several hypotheses have been proposed, including hypersensitivity reactions to infectious agents (such as Toxoplasma gondii, Epstein–Barr virus, varicella‐zoster virus, parvovirus B19, cytomegalovirus, HIV, group A streptococcus, *Staphylococcus aureus* , and *Mycoplasma pneumoniae* ), immune complex‐mediated hypersensitivity vasculitis, and T‐cell dyscrasia, including possible association with cutaneous T‐cell lymphoma [11, 12]. Since PLEVA is generally a benign and self‐limiting disorder, clinical observation without pharmacological treatment may be appropriate in mild and localized cases [1]. However, more extensive, persistent, or symptomatic cases may require therapy. Reported treatments include topical corticosteroids, oral antibiotics (such as macrolides, tetracyclines, and dapsone), narrow‐band UVB phototherapy, and immunomodulatory agents [13]. Tacrolimus ointment has also shown efficacy in selected cases, with reported complete remission in PLC patients [14]. The frequent association of PL with infectious episodes and vaccinations (including MMR, influenza, and tetanus‐diphtheria vaccines) supports the hypothesis of an aberrant immune response to external antigenic stimuli in genetically predisposed individuals [1]. In addition, several cases of PLEVA following COVID‐19 vaccination have been reported in adults [15, 16, 17, 18, 19], and are summarized in Table 1.

**TABLE 1:** Comparison between Patient 1 and the cases of COVID‐19 vaccine‐associated PLEVA reported in the literature.

	Authors (year)				
	Mäkilä et al. (2022) [15]	Sechi et al. (2021) [18]	Sernicola et al. (2022) [16]	Palmen et al. (2022) [19]	Our Patient 1
Age	21 years	70 years	31 years	81 years	6 years
Gender	F	M	F	M	M
Association between PLEVA and COVID‐19 vaccine	PLEVA after SARS‐CoV‐2 infection. Exacerbation 10 days after the 2nd dose of COVID‐19 vaccine (6 weeks after phototherapy treatment).	5 days after first vaccination with BNT162b2 (Pfizer‐BioNTech)	10 days after first vaccination with BNT162b2 (Pfizer‐BioNTech)	9 days after second vaccination with BNT162b2 (Pfizer‐BioNTech)	7 days after first dose of COVID‐19 vaccine BNT162b2 (Pfizer‐BioNTech)

In the Italian pediatric population, a few cases (about ten) of papulo‐purpuric rash with characteristics of PLEVA following SARS‐CoV‐2 infection have been ascertained, but none has been described related to COVID‐19 vaccination [20]. Currently, there is no classification of skin reactions to COVID‐19 vaccine, and their clinical presentation is poorly detailed. The available data are included in the international registry of skin manifestations from SARS‐CoV‐2 (www.aad.org/covidregistry) [21]. The most frequently observed events (similarly to what happens for other vaccinations, even for those recently introduced) [22, 23] are urticaria, local reactions at the injection site, and morbilliform skin eruptions. Our Patient 1 may represent, to the best of our knowledge, the first reported pediatric case clinically consistent with PLEVA occurring after BNT162b2 (Pfizer‐BioNTech) COVID‐19 vaccination. Serological findings demonstrating vaccine‐induced antibody response, together with the temporal association, support a possible immunologically mediated relationship between vaccination and disease onset in this case. The second clinical case highlights a possible, although rare, association between PLEVA and GAS infection. In this context, antibiotic therapy for streptococcal pharyngotonsillitis was appropriately administered, leading to rapid resolution of both systemic and cutaneous manifestations. Streptococcal infection may act as an immunological trigger or modifier in susceptible individuals, similarly to other immune‐mediated post‐infectious conditions [24], like acute rheumatic fever or glomerulonephritis. In the cohort studied by Elbendary et al., streptococcal infection seems to trigger immunological reactions that could cause or reactivate PLC in susceptible subjects. Actually, azithromycin, in addition to its antimicrobial activity, may exert immunomodulatory effects that contribute to clinical improvement in PL, further supporting the hypothesis that modulation of immune response rather than pathogen eradication may be central in disease control [24]. In both cases, careful clinical evaluation and exclusion of systemic involvement allowed identification of two distinct potential triggers of PLEVA: vaccination in the first case and bacterial infection in the second. Both patients showed a benign and self‐limiting course, supporting conservative therapeutic management (Table 2).

**TABLE 2:** Comparison of clinical features, diagnostic tests, treatment, and evolution between our patients.

	Patient 1	Patient 2
Age	6 years	5 years
Gender	M	M
Skin lesions	Non‐pruritic papules, with a purpuric appearance, some surmounted by a central scaly crust, spread to the trunk, and upper limbs	Micro‐papular lesions with a purpuric appearance, slightly itchy, some with necrotic evolution, spread to the trunk, limbs, and folds
Extra‐cutaneous manifestations	—	Hyperemic pharynx with tonsillar exudate
Diagnostic tests	Normal blood tests, negative infectious disease screening	Normal blood tests, negative infectious disease screening except for throat swab (positive for GAS)
Correlation with vaccine/infectious agent	COVID‐19 vaccine (*mRNA Pfizer‐BionTech*)	GAS infection
Treatment	Topical hydrocortisone Azithromycin orally 250 mg/day for 5 days every other week (3 cycles)	Topical hydrocortisone Oral amoxicillin 50 mg/kg twice/day for 7 days
Clinical evolution	Partial regression of lesions after 6 weeks	Definitive regression of the lesions after approximately 1 month

Although PLEVA after COVID‐19 vaccine is a rare event, the similarities with the adult cases reported in the literature could support its inclusion among the skin manifestations associated with such vaccine. The analysis of the case secondary to streptococcal disease could, conversely, open new perspectives for the understanding of the pathogenesis of PL, suggesting a role for this infection as an immunological trigger.

Despite the absence of histopathological confirmation, which represents a limitation of this report, the overall clinical presentation and follow‐up of our patients strongly supported the diagnosis of PLEVA. Although pityriasis lichenoides is an uncommon condition, pediatricians should consider it in the differential diagnosis of papulo‐necrotic eruptions, particularly in specific infectious or post‐vaccination contexts. Accurate anamnesis and careful clinical evaluation remain, then, essential for diagnosis and appropriate management.

## Author Contributions

**Gregorio Serra:** conceptualization. **Deborah Bacile:** writing – original draft. **Elisabetta Di Leto:** data curation. **Ornella Iacono Fullone:** data curation, formal analysis. **Chiara Martorana:** data curation, formal analysis. **Ilaria Pirrone:** data curation, formal analysis. **Mario Tumminello:** supervision. **Giovanni Corsello:** conceptualization, writing – review and editing.

## Funding

The authors have nothing to report.

## Ethics Statement

The study was approved by the Mother and Child Department of the University of Palermo (Palermo, Italy). All procedures performed in this study were in accordance with the ethical standards of the institutional and national research committee, and with the 1964 Helsinki declaration and its later amendments or comparable ethical standards.

## Consent

Written informed consent was obtained from both patients' parents.

## Conflicts of Interest

The authors declare no conflicts of interest.

## Data Availability

The data that support the findings of this study are available from the corresponding author upon reasonable request.
